# A Rare Case of Extra-abdominal Desmoid-type Fibromatosis Arising from the Popliteal Fossa

**DOI:** 10.7759/cureus.3651

**Published:** 2018-11-28

**Authors:** Mantu Jain, Sudhanshu S Das, Amrit Gantaguru, Ritesh Panda, Sudarsan Behera

**Affiliations:** 1 Orthopedics, All India Institute of Medical Sciences, Bhubaneswar, IND; 2 Orthopedics, All India Institute of Medical Sciences, Bhubaneswar , IND; 3 Plastic Surgery, All India Institute of Medical Sciences, Bhubaneswar, IND; 4 Orthopaedics, All India Institute of Medical Sciences, Bhubaneswar, IND

**Keywords:** desmoid type fibromatosis, popliteal fossa

## Abstract

Desmoids are rare soft-tissue tumors of the abdominal wall that may sporadically occur extra-abdominally. It manifests as clonal fibroblastic proliferation with an infiltrative tendency and capacity to recur without metastasizing. An adolescent male presented with a gradually increasing globular, non-tender, firm, non-pulsatile swelling (8 × 5 × 3 cm^3^) in the left popliteal fossa that had been present for five months. Following thorough investigation with imaging and Tru-cut biopsy, finally, an excisional biopsy was done. Histopathological examination confirmed a desmoid tumor, and the patient received adjuvant radiotherapy. At the one-year postoperative follow-up, there was no recurrence; the patient had been explained the prognosis. This case highlights a rare site of an extra-abdominal desmoid but with classical clinical presentation, imaging, intraoperative, and histopathological findings. Awareness and knowledge of this entity are of paramount importance for clinical practitioners.

## Introduction

Muller first coined the term desmoids; these tumors have been identified to arise from fascial and musculo-aponeurotic tissue, mostly in the abdominal wall [1]. However, sporadic desmoids occur in almost any part of the body including the head and neck, chest wall, trunk, and extremities [1, 2]. An association of desmoids with Gardner and other familial syndromes has been reported [3]. The clinical presentation of desmoids is variable and, although the tumor is more common during adolescence and has a female predominance, it has been reported in infants as well in the elderly (>65 years) [4]. The mass grows relatively rapidly whereas remaining non-tender; however, it has a tendency to infiltrate surrounding structures and has a high probability of recurrence even after an apparent microscopically margin-negative resection (R0) surgical resection [5].

## Case presentation

**Figure 1:**
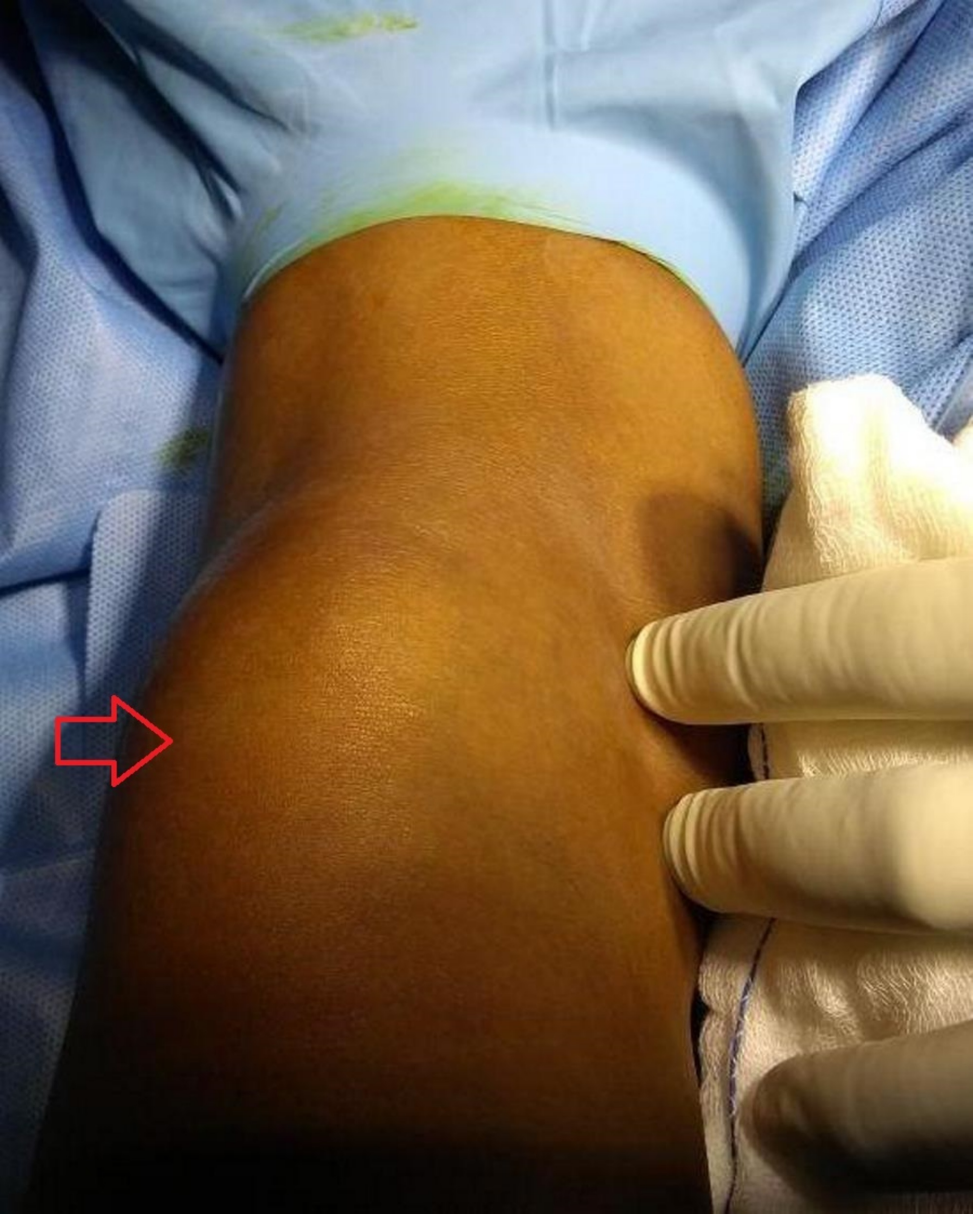
Clinical appearance of the mass (red arrow) in the popliteal fossa.

A 16-year-old male presented to the orthopedic outpatient department of our hospital with a gradually increasing, globular, non-tender, non-pulsatile, firm swelling that measured approximately 8 × 5 ×3 cm^3^ and was situated in the left popliteal fossa since five months (Figure 1).

**Figure 2:**
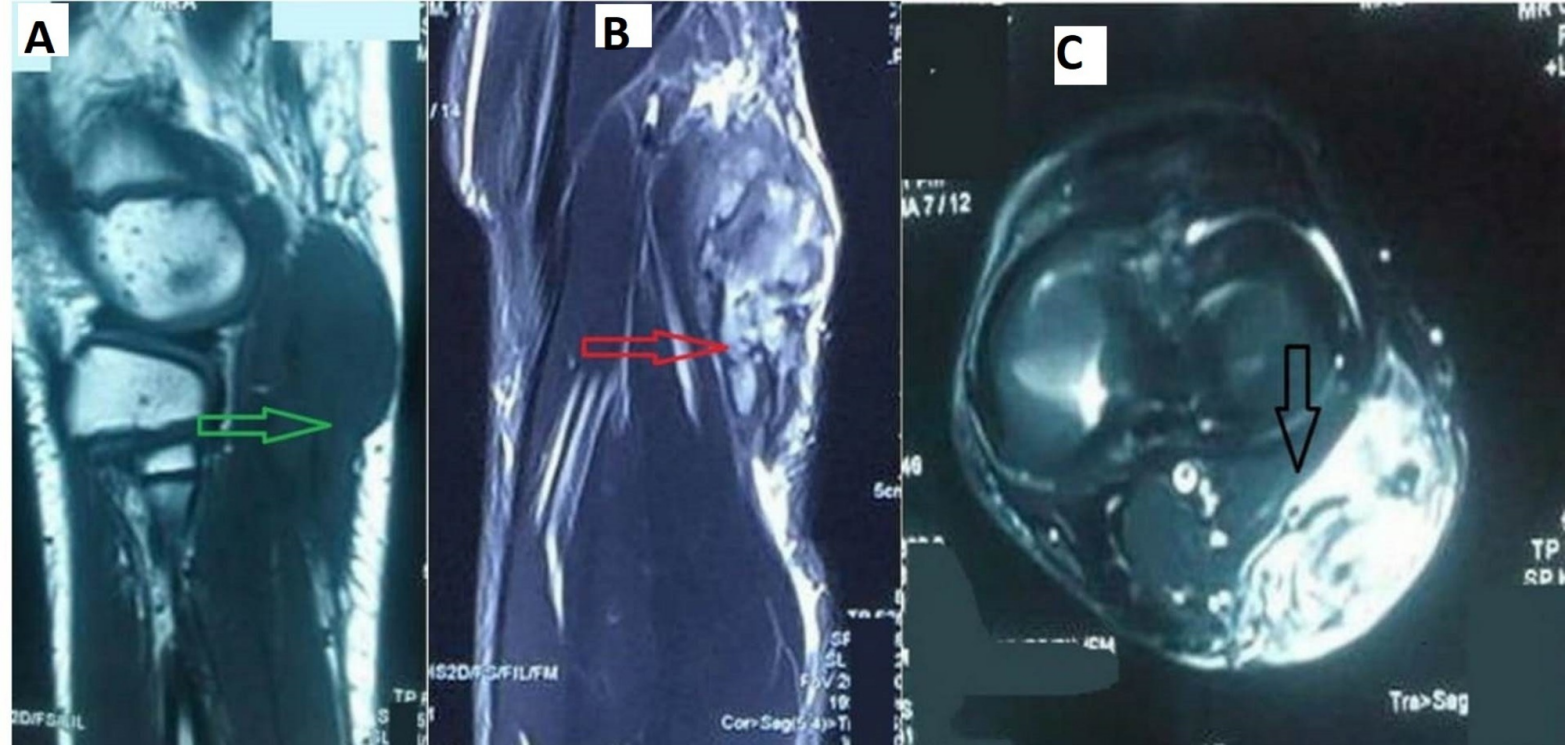
Magnetic resonance imaging showing an ill-defined, lobulated mass that is A) hyperintense on T2-weighted imaging (green arrow), B) hypointense on T1-weighted (red arrow) and C) STIR sequence with contrast enhancement adjacent to the lateral head of the gastrocnemius (black arrow). STIR: Short-TI Inversion Recovery.

The swelling was attached to underlying structures, but was free from skin, and there was no distal neurovascular deficit. A plain X-ray of the knee was unremarkable. Magnetic resonance imaging (MRI) of the lesion revealed an ill-defined, lobulated, soft-tissue, space-occupying lesion in the lateral aspect of the calf adjacent to the lateral head of the gastrocnemius that was hypointense on the T1-weighted image and hyperintense on the T2-weighted image, with multiple ill-defined T2-weighted signal voids within the lesion that gave an impression of hemangioma (Figures 2A-2C).

**Figure 3:**
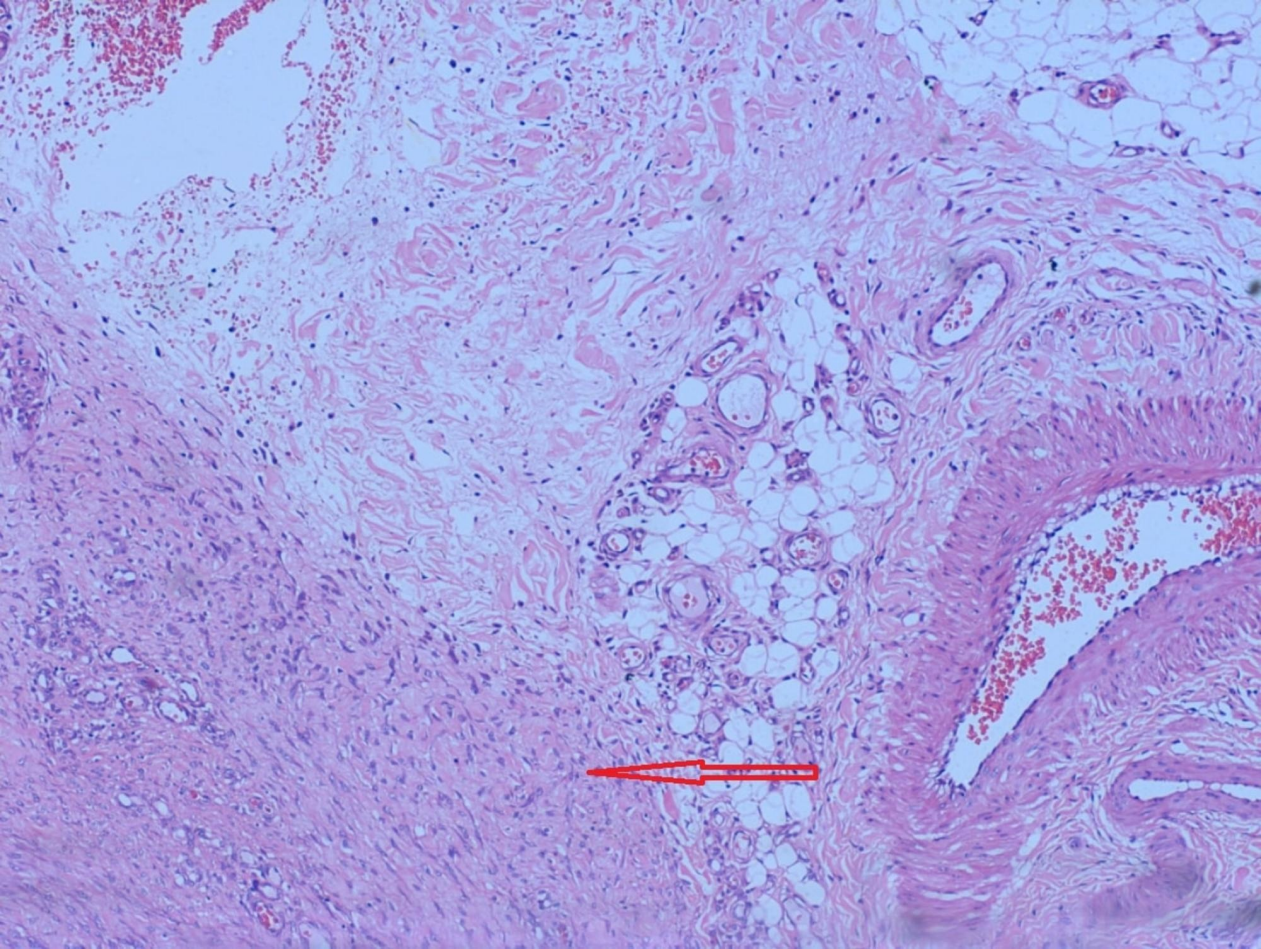
Photomicrograph of the surgical specimen showing plenty of fibroblasts (red arrow) against the collagen background that are infiltrating normal tissue.

Tru-cut biopsy showed spindle cells arranged in clusters and scattered singly in a hemorrhagic background with no evidence of malignancy, suggesting a benign spindle cell tumor. Histopathological examination with Hematoxylin–Eosin staining revealed plenty of fibroblasts spread against a background of collagen as well as infiltration of the adjoining healthy tissue that led to a microscopic diagnosis of desmoid fibromatosis (Figure 3).

Blood investigations as part of the routine preoperative workup showed normal results. Clinically, desmoid tumor mimics other soft-tissue tumors such as leiomyoma, rhabdomyoma, nerve sheath tumor, as well as vascular and perivascular tumors. Common histological differential diagnoses include fibrosarcoma, spindle cell tumor, epithelioid tumor, and pleomorphic tumor.

**Figure 4:**
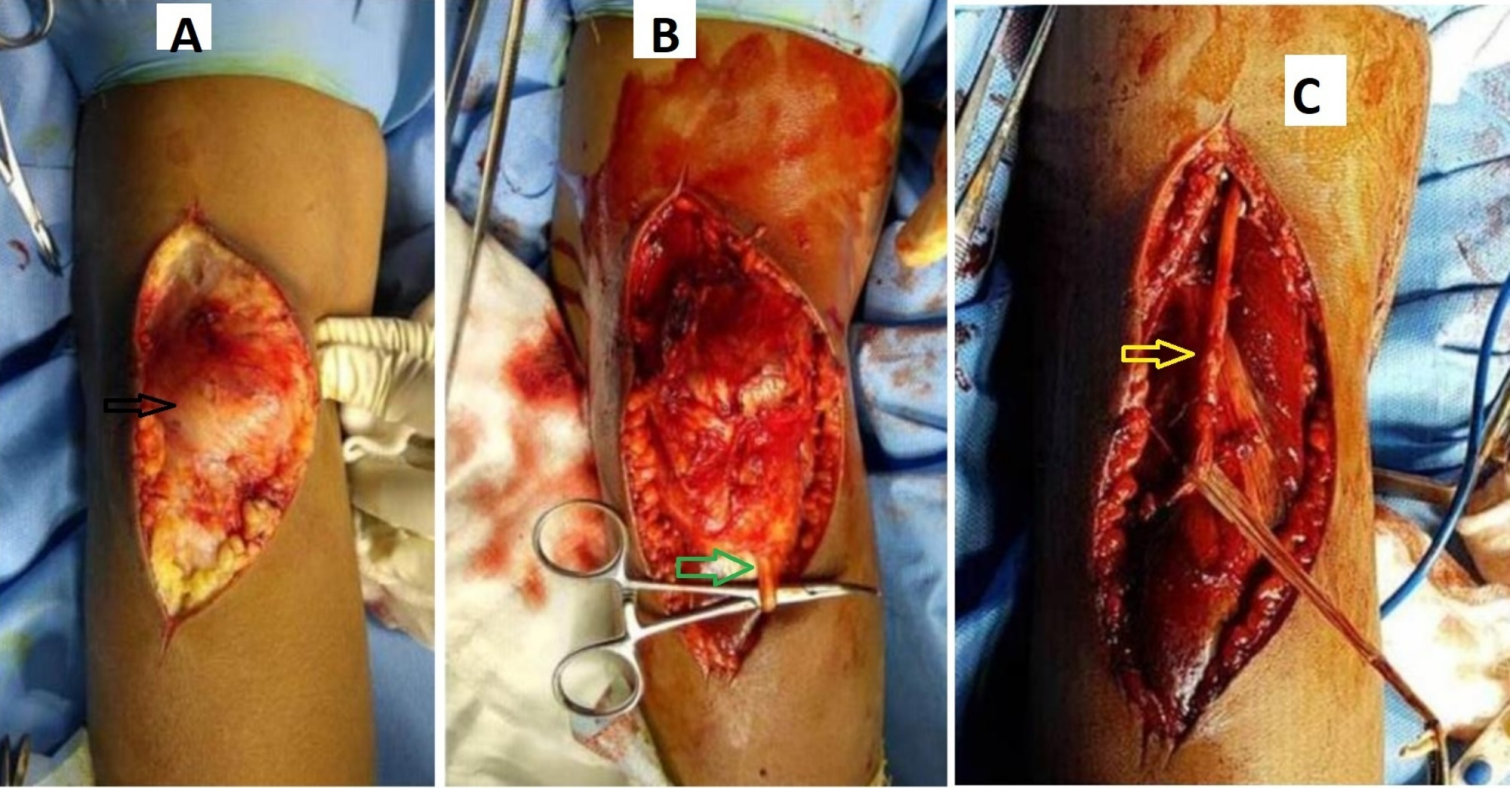
Intraoperative image showing A) the tumor deep to fascia (black arrow) B) involving the sural nerve (green arrow), and C) involving the common peroneal nerve (yellow arrow) as well, which was dissected out completely.

Under general anesthesia, the patient was positioned prone and a J-shaped lazy posterior incision was made. The tumor was situated deep to the fascia although it involved it. On palpation, it was firm, partly smooth, and seemed to arise from the deep fascia and muscle without any clearly defined planes; it was located adjacent to the lateral head of the gastrocnemius. The mass did not engulf any major vascular structures in the popliteal fossa although the common peroneal nerve and the medial cutaneous sural nerve traversed it. The sural nerve was sacrificed and the common peroneal dissected out carefully; the mass was then removed, and the specimen in toto was sent for histopathological study (Figure 4).

The wound was closed in layers. There were no immediate postoperative complications. The dorsiflexors of the ankle and toes were well preserved. The patient was discharged on tenth postoperative day and received adjuvant radiotherapy after three weeks to minimize any chance of recurrence. At the one-year follow-up, the patient had no radiological or clinical evidence of recurrence, and has been advised regular follow-ups every six months.

## Discussion

With an incidence of approximately three to four cases per million, desmoids comprise only 3% of all soft-tissue tumors [6]. The typical site of a desmoid is the abdominal wall, although it sometimes occurs intra-abdominally and involves the bowel and mesentery [7]. Extra-abdominal sites of desmoid occurrence include the chest wall, trunk, neck, shoulder girdle, and inguinal region. The popliteal fossa is an extremely rare location for desmoid tumors, with only a few cases reported in the literature [7, 8]. The pathogenesis of desmoid tumors is not completely understood. Risk factors include female sex, and its high incidence during pregnancy suggests a causal association with estrogen levels [9]. Furthermore, genetic susceptibility through β-catenin deregulation (CTNNB1 encodes for β-catenin) has been proved [10]. Desmoid tumors have been associated with Gardner’s and other familial syndromes. Diagnosing these tumors is a real challenge for the treating surgeons because they are rare and can occur at unusual extra-abdominal locations. McKinnon et al. suggested that only half of all desmoid cases can be correctly diagnosed preoperatively [11]. Moreover, MRI findings may be asynchronous from clinical findings. Kaygin et al. reported a desmoid tumor of the popliteal fossa that appeared to be a vascular mass on MRI [7]. Similarly, even in the presented case, the tumor presented as a non-pulsatile, non-tender mass with a radiological impression that favored a diagnosis of vascular malformation. Macroscopically, desmoid-type fibromatosis is a rubbery grayish white lesion, fibrotic, with an irregular scarred appearance, without a capsule, and can grow to large sizes and infiltrate nerves in the vicinity of the tumor – all of which was well-evidenced in the present case. The Tru-cut biopsy suggested a differential diagnosis of a benign spindle tumor; however, histological examination revealed a dense, collagenous lesion with intertwining bundles of spindle cells that were uniform, lacked mitotic activity, but infiltrated the surrounding tissue. No necrosis or cellular anaplasia was present. There is no consensus with regard to the best treatment for a desmoid tumor [6]. Complete surgical excision should be the aim of treatment whenever possible, together with adjuvant radiotherapy/chemotherapy. Despite best efforts, the recurrence rate remains high, particularly in cases that undergo subtotal resection [5]. Diaz et al. reported a recurrent desmoid of the popliteal fossa with vascular compromise [8].

## Conclusions

The popliteal fossa is an unusual location for an extra-abdominal desmoid-type fibromatosis. Diagnosing this tumor is challenging and histopathological study is necessary for a final diagnosis. Adjuvant radiotherapy minimizes chances of tumor recurrence.
